# Supplementary figures and images for: Synaptic plasticity onto inhibitory neurons as a mechanism for ocular dominance plasticity
Source: PLoS Comput Biol. 2019 Mar 12;15(3):e1006834. doi: 10.1371/journal.pcbi.1006834 (PMC6430420; doi:10.1371/journal.pcbi.1006834)

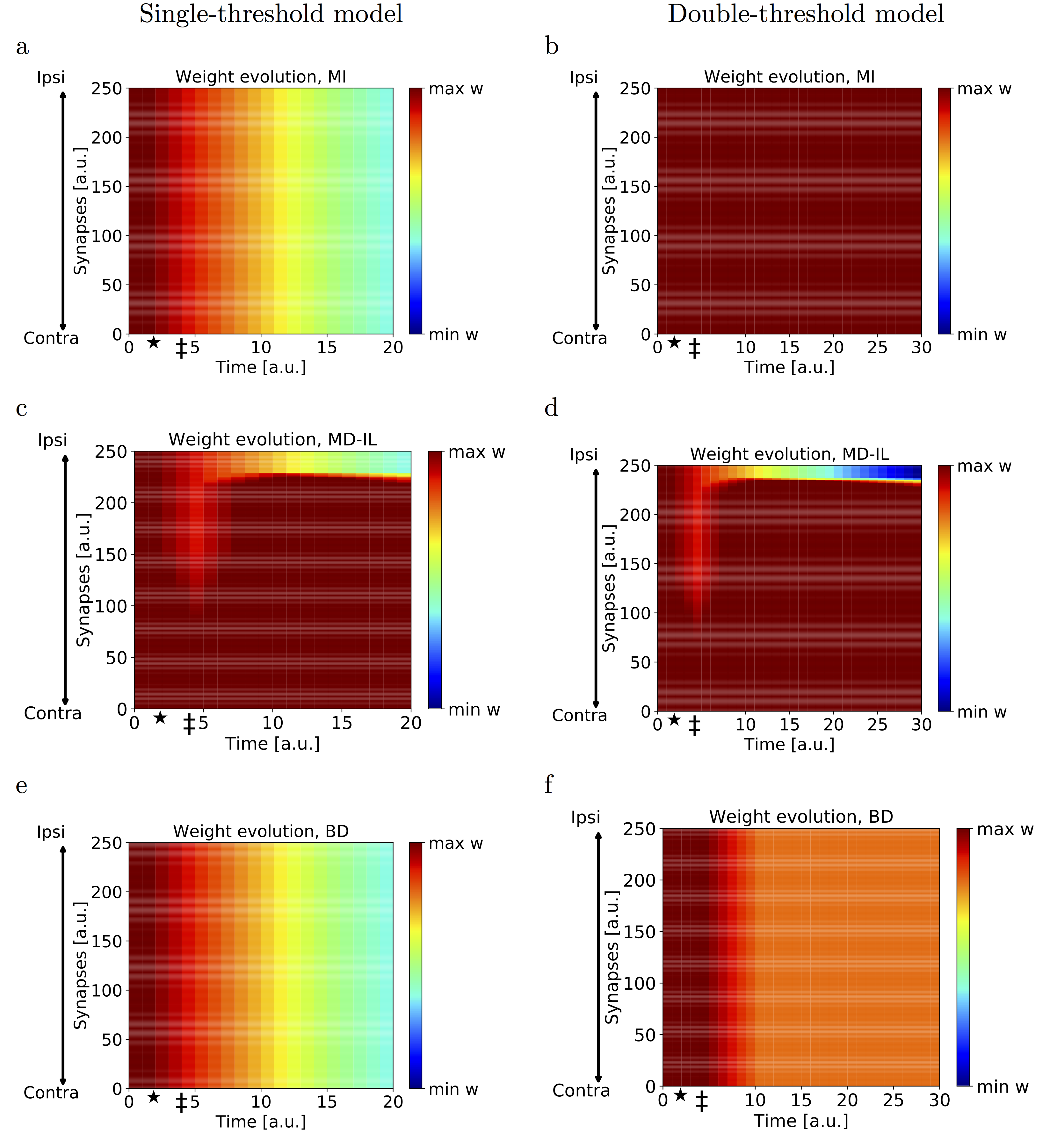

Supplement: S1 Fig — (a), (c), (e) Evolution of synaptic weights over time for the single-threshold model, in the cases of MI, MD-IL and MD-BD respectively. The star denotes the onset of deprivation, the double dagger denotes the onset of reduced inhibition. (b), (d), (f) Evolution of synaptic weights over time for the double-threshold model, in the cases of MI, MD-IL and BD. The star denotes the onset of deprivation, the double dagger denotes the onset of reduced inhibition. (TIFF) [file pcbi.1006834.s001.tiff]

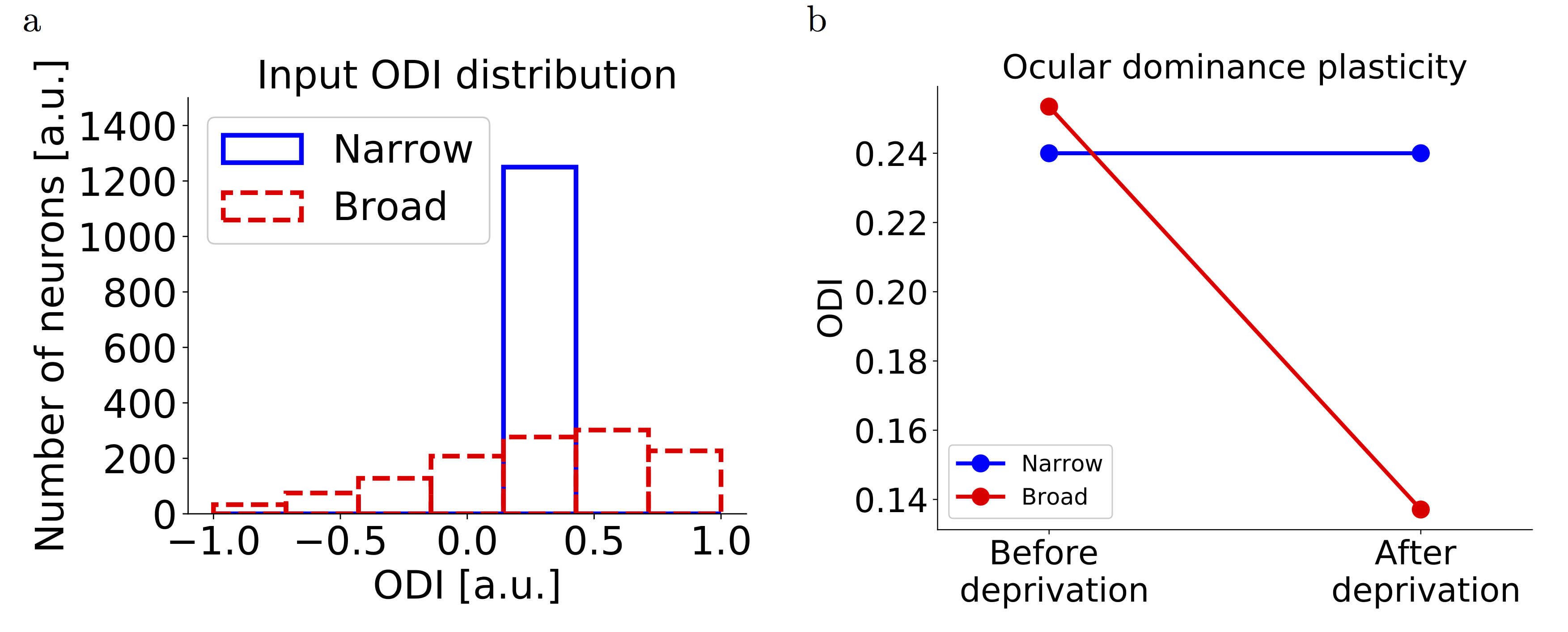

Supplement: S2 Fig — (a) A layer II/III neuron receives inputs with either a narrow ODI distribution (blue) or a broad distribution (red). (b) Layer II/III neurons with similar ODI index only show an OD shift after MD when the inputs have a broad ODI distribution. (TIFF) [file pcbi.1006834.s002.tiff]

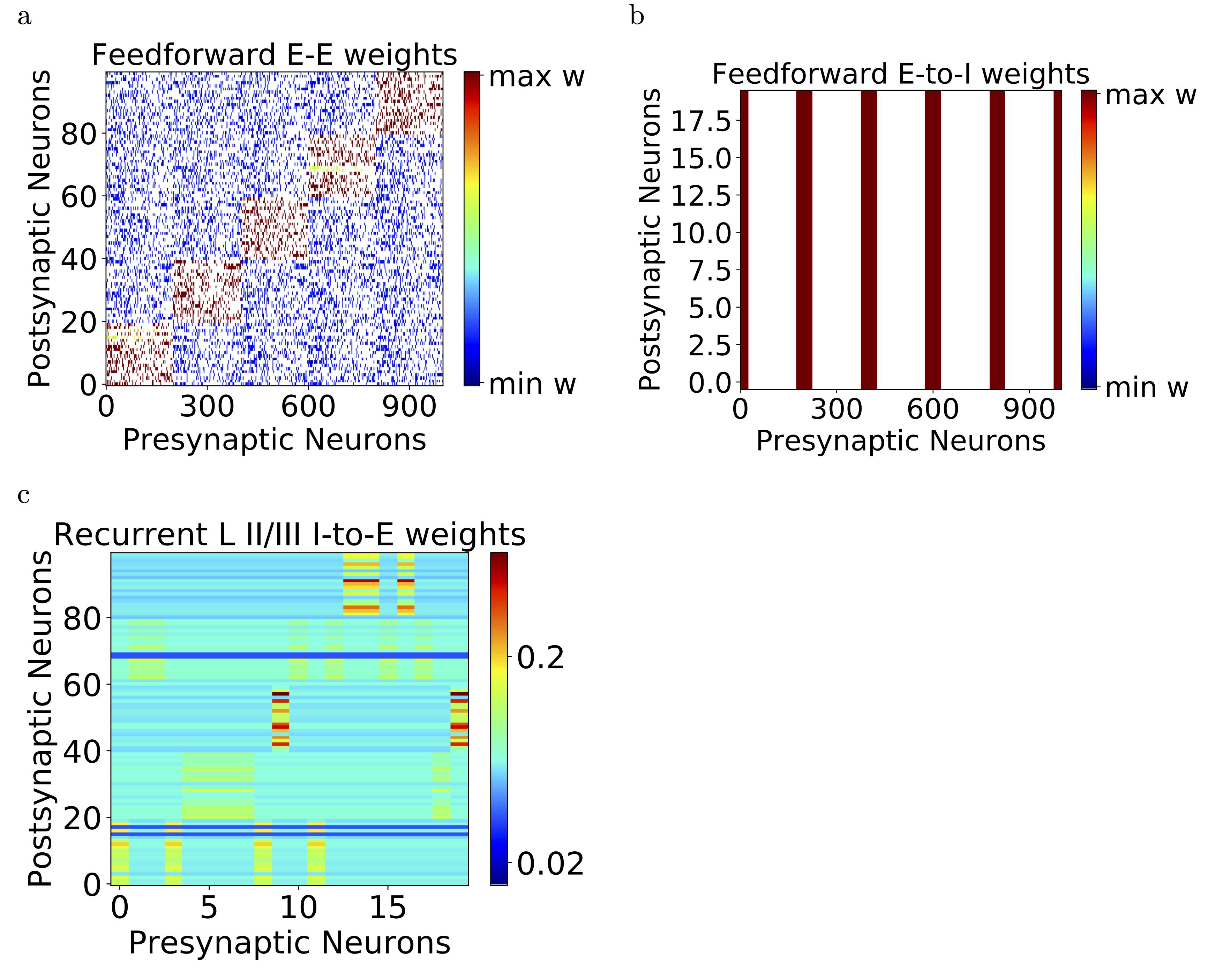

Supplement: S3 Fig — (a) Feedforward E-to-E weights are specific, only synapses from one input group are strong, while other feedforward inputs are weak. Only 50 feedforward connections per input group are made, white denotes no connection (see Methods). (b) Feedforward E-to-I weights are unspecific, synapses from all input groups (200 neurons) are at the maximum bound. Only 50 feedforward connections per input group are made, white denotes no connection (see Methods). (c) Recurrent I-to-E weights after the first learning phase. (TIFF) [file pcbi.1006834.s003.tiff]

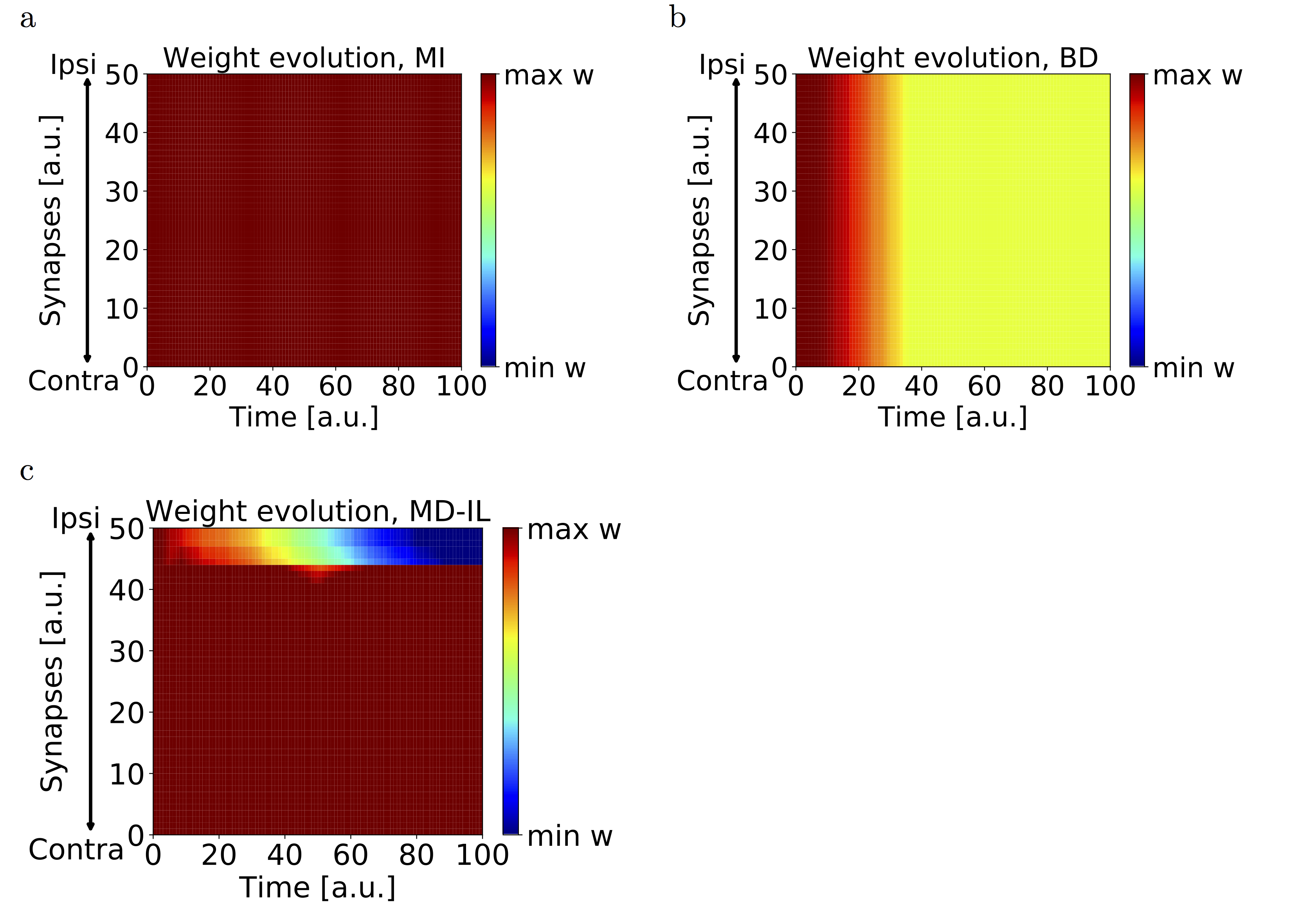

Supplement: S4 Fig — (a), (b), (c) Evolution of synaptic weights over time for one neuron in the network, in the cases of MI, BD, MD-IL. (TIFF) [file pcbi.1006834.s004.tiff]

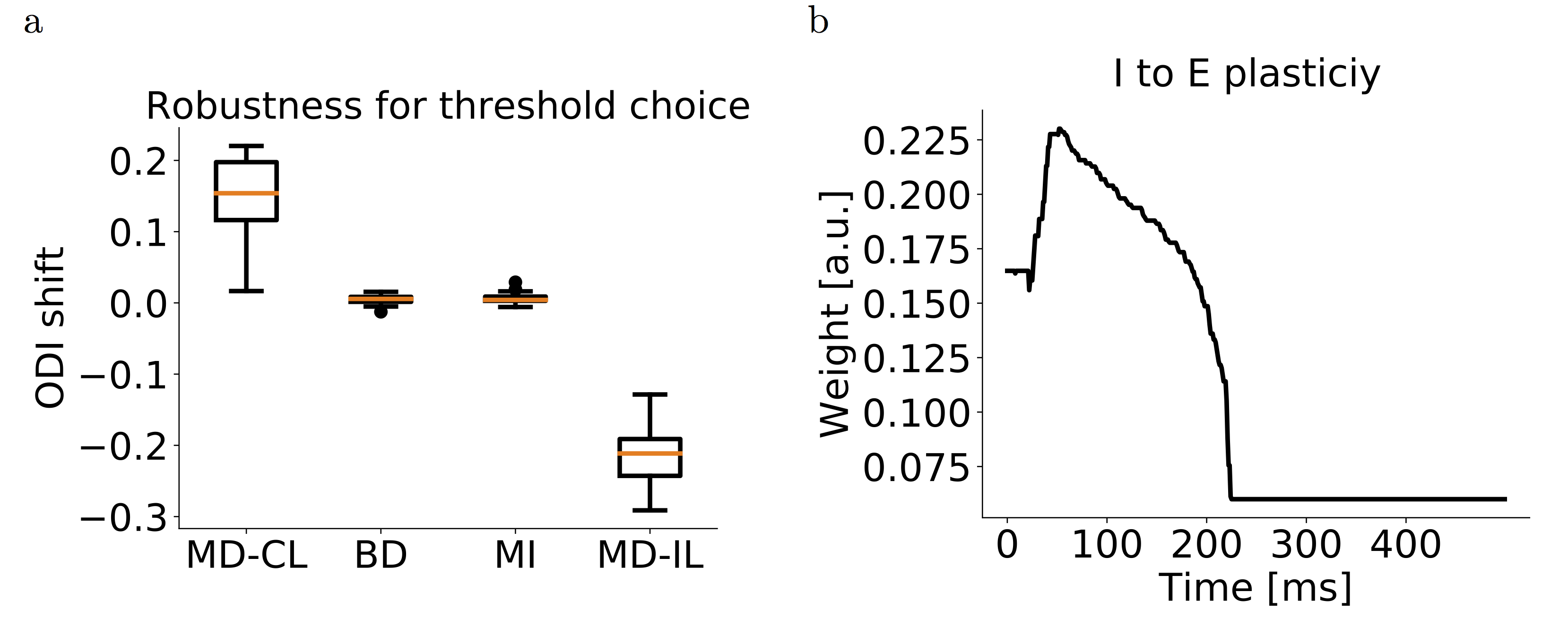

Supplement: S5 Fig — (a) Our results are robust against changes in threshold values. The boxplots show the distribution of population ODI shifts after 50 simulations for all types of deprivation. In each simulation, a random normal value for θH and θL is chosen with as mean the usual values (see Methods) and standard deviation 5% of these values. (b) Evolution of the I-to-E weights after deprivation. Potentiation is observed to partially counteract the reduction of inhibition in E-to-I connections, followed by depression to a minimum bound when the inhibition recovers. (TIFF) [file pcbi.1006834.s005.tiff]

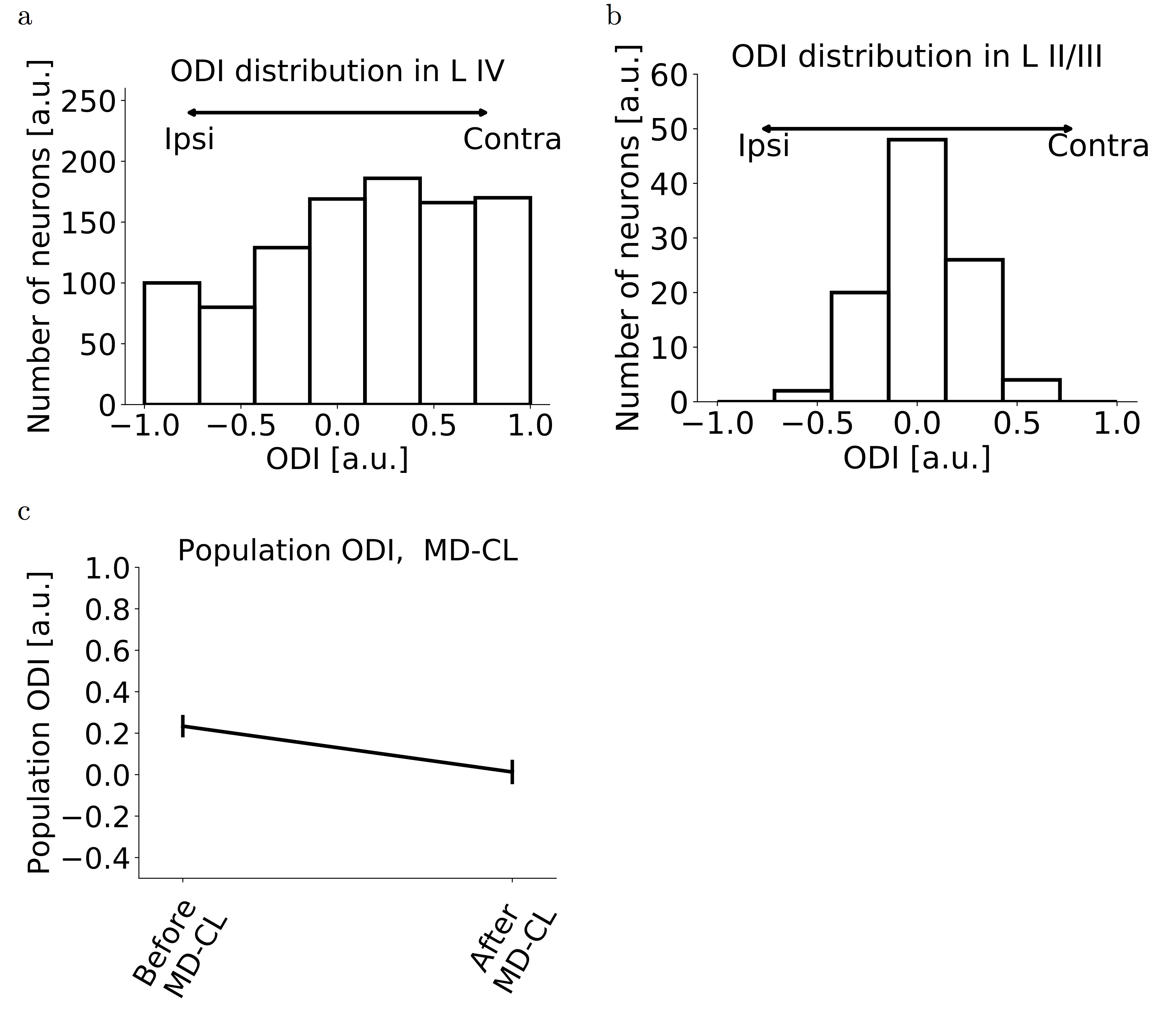

Supplement: S6 Fig — (a) Example of ODI distribution in layer IV. (b) Randomly sampling 50 connections from the layer IV population leads to a narrow distribution in layer II/III. (c) Recurrent layer II/III connections are not crucial in our model. Similar results are obtained by reducing the θH and the θL by 1. (TIFF) [file pcbi.1006834.s006.tiff]
